# The Identification and Characterization of Chitotriosidase Activity in Pancreatin from Porcine Pancreas

**DOI:** 10.3390/molecules18032978

**Published:** 2013-03-04

**Authors:** Chia-Rui Shen, Chao-Lin Liu, Hsiao-Ping Lee, Jeen-Kuan Chen

**Affiliations:** 1 Department of Medical Biotechnology and Laboratory Science, College of Medicine, Chang Gung University, 259 Wen-Hwa 1st Road, Kweishan, Taoyuan 33302, Taiwan; E-Mail: crshen@mail.cgu.edu.tw; 2 Department of Chemical Engineering and Graduate School of Biochemical Engineering, Ming Chi University of Technology, 84 Gung-Juan Road, Taishan, Taipei 24301, Taiwan; 3 Department of Environment and Biotechnology, Refining & Manufacturing Research Institute, CPC Corporation, 217 Min-Sheng S. Rd, Chiayi 60051, Taiwan; E-Mail: 078638@cpc.com.tw

**Keywords:** chitin, chitosan, oligosaccharides, pancreatin, chitotriosidase

## Abstract

The versatile oligosaccharide biopolymers, chitin and chitosan, are typically produced using enzymatic processes. However, these processes are usually costly because chitinases and chitosanases are available in limited quantities. Fortunately, a number of commercial enzymes can hydrolyze chitin and chitosan to produce long chain chitin or chitosan oligosaccharides. Here, a platform to screen for enzymes with chitinase and chitosanase activities using a single gel with glycol chitin or glycol chitosan as a substrate was applied. SDS-resistant chitinase and chitosanase activities were observed for pancreatin. Its chitotriosidase had an optimal hydrolysis pH of 4 in the substrate specificity assay. This activity was thermally unstable, but independent of 2-mercaptoethanol. This is the first time a chitotriosidase has been identified in the hog. This finding suggests that oligochitosaccharides can be mass-produced inexpensively using pancreatin.

## 1. Introduction

Chitin, an insoluble linear (1→4)-β-linked homopolymer of *N*-acetyl-d-glucosamine (GlcNAc), is the most abundant polysaccharide in Nature and is distributed ubiquitously. Chitins are found in arachnids, the cell walls of most fungi, insect exoskeletons, crustacean shells, and in invertebrates [1]. They are also synthesized by some bacteria as an extracellular polymer [2,3]. Approximately 3.7 × 10^4^ tons are produced by marine invertebrates alone [4], and the annual rate of steady-state of chitin synthesis is approximately 10^10^ to 10^11^ tons worldwide [5]. Most biological materials composed of chitin eventually become biological solid waste.

Chitin and its derivatives, including polysaccharides, oligosaccharides and monosaccharides, have been used for enzyme immobilization and waste treatment since the 1970s. In the last two decades, these compounds have been the subject of intense interest once their versatile biological functions and activities were identified [6,7]. They have been shown to play roles in plant organogenesis and invertebrate embryogenesis [8]. The addition of chitin to soil can reduce the populations of fungal plant pathogens [9] and plant parasitic nematodes by increasing the populations of chitinolytic bacteria (*i.e.*, bacteria with chitinases) [10,11,12]. Chitin oligosaccharides have also been investigated in efforts to enhance immune system function in host animals [13,14] and may display tumoricidal properties [15,16,17,18]. In addition, several chitin derivatives have antimicrobial and cholesterol-reduction activities [7]. Because chitin derivatives are biodegradable, histocompatible, and nontoxic, they are often used in biomedical materials, such as artificial joints. Currently, they are usually obtained by either chemical cleavage or enzymatic degradation. However, the products of these chemical reactions are short chain molecules [19]. In addition, chitin hydrolytic reaction and purification processes typically produce environmental pollutants. Therefore, enzymatic procedures are preferred because chitinases or chitosanases produce more homogeneous oligosaccharides with higher molecular weights. However, these reactions are limited due to the poor stability, low activities and high costs of those enzymes.

Based on their catalytic mechanisms and products, chitinases are categorized as endochitinases (EC 3.2.1.14), chitobiosidases (EC 3.2.1.30), and exochitinases (EC 3.2.1.52) [20,21]. The enzymatic degradation of chitin is similar in prokaryotes and eukaryotes. The first step, which usually occurs in microbes and plays a key role in the solubilization and mineralization of chitin, is the random hydrolysis of the glycosidic bonds between GlcNAc residues by endochitinases to yield chitin oligomers. This is followed by further digestion from the nonreducing ends by the monomer-producing exochitinases known as β-*N*-acetylhexosaminidases. In some organisms, chitobiosidases act between these two steps to release GlcNAc dimers from the nonreducing ends to facilitate the degradation [1,22]. 

Chitinases are distributed among numerous organisms from all animal kingdoms, including viruses, bacteria, higher plants and animals [1,23]. In organisms with endogenous chitin, the control of chitinase digestion or chitin deformation must be effective [24], and the chitinases produced are presumably required for cell wall or exoskeleton morphogenesis [25]. Plants and mammals, however, do not contain chitin, and the roles of the chitinases in these organisms remain enigmatic. It has been postulated that plants produce chitinases to protect themselves from chitin-containing parasites or invaders [26]. In mammals, a few chitinases and chitinase-like proteins have been identified in the immune cells, respiratory tracts and gastrointestinal systems of rats, horses and humans [27,28,29,30]. These enzymes are involved in inflammation and tissue remodeling [31]. In addition, some commercial enzymes with unexpected chitosanolytic and chitinolytic activities have been identified. These include glycanases, cellulases, amylases, dextranases, hemicellulases and pectinases. Using these enzymes, chitin and chitosan derivatives can be obtained at ambient temperature [32].

Chitinolytic or chitosanolytic activity can be detected by enzymatic hydrolysis in a solution or gel. In solution assays, the activity can be determined using the products, reducing sugars, fluorophores or chromophores [33]. The drawback to this type of assay is that the activity could potentially be due to contaminants. Chitinolytic or chitosanolytic activities can also be observed using in-gel activity staining; one polyacrylamide gel with SDS (SDS-PAGE) is stained using Coomassie Brilliant Blue R-250 (CBB R250) to allow protein localization, while a second gel, without SDS, is stained with Calcofluor white M2R to allow chitinase detection using UV illumination [34,35,36]. However, there are several disadvantages to this technique. Namely, this assay requires a large quantity of sample because two gels are applied, and the molecular weights are usually poorly estimated. In addition, the use of UV illumination to observe activity zones may be dangerous. Recently, an in-gel chitinase assay was developed. Using this assay, chitinase can be identified in a single SDS-PAGE gel containing glycol chitin and stained with CBB R250 [21,33,37].

The aim of this study was to identify low-cost chitinases that produce oligosaccharides of chitin derivatives. In this study, a screening platform was introduced to evaluate enzymes active on chitin or chitosan. Using this technique, pancreatin was found to have SDS-resistant (SDSR) chitinase and chitosan activities. Further substrate assays showed that the pancreatin possessed chitotriosidase activity.

## 2. Results and Discussion

### 2.1. Sensitivity of In-gel Chitinase and Chitosanase Assays

**Figure 1:**
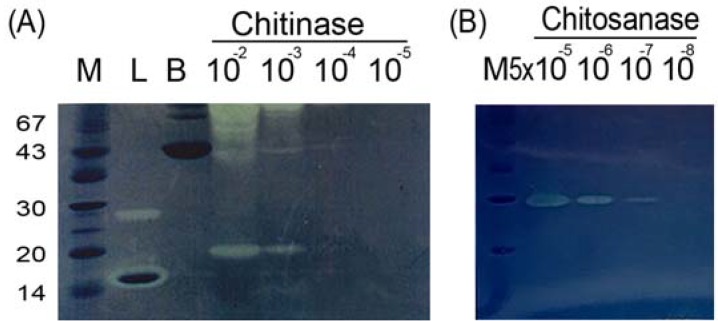
The sensitivities of in-gel chitinase assay using serial dilution of units of *Streptomyces griseus* chitinase (**A**) and chitosanase assay using *Streptomyces* sp. chitosanase (**B**). Lane M, protein markers; Lane L, lysozyme, as positive control; Lane B, bovine serum albumin, as negative control.

Chito-oligosaccharides are versatile biomolecules. Long-chain oligomers can be obtained from chitin or chitosan that has been digested by enzymes. For commercial applications, enzymatic processes are expensive due to both the limited availability and the low stability of chitinases and chitosanases. The feasibility of using degraded chitin and chitosan as substrates for low-cost enzymes in aqueous solution was evaluated. It has been reported that CBB R250, an acidic dye, can adsorb chitin and chitosan. The adsorption capacity of CBB R250 is proportional to the degree of deacetylation of the chitin or chitosan [38]. For the in-gel assays, a glycol chitosan or glycol chitin substrate with a known degree of acetylation was cast into SDS-PAGE gels. The sensitivity of the glycol chitin assay was 10^−4^ U (Figure 1A), while that of the glycol chitosan assay was 5 × 10^−7^ U. (Figure 1B). The detection sensitivity was maintained when chitosans with various molecular weights, representing different levels of polymerization, were used for the in-gel glycol chitosan assays. These results indicated that the sensitivity of the assay was independent of the degree of polymerization but dependent upon the degree of acetylation.

### 2.2. Unusual Susceptibilities of Chitin and Chitosan to Enzymatic Hydrolysis

**Figure 2:**
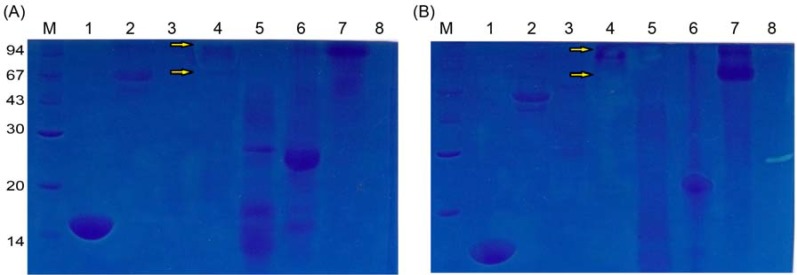
Unusual susceptibilities of chitin (**A**) and chitosan (**B**) to enzymatic hydrolysis demonstrated using the in-gel assay. Lane M, protein markers; Lane 1, lysozyme; Lane 2, cellulose; Lane 3, biopectinase; Lane 4, pancreatin; Lane 5, bromelain; Lane 6, papain; Lane 7, lactase; Lane 8, chitosanase.

Chitin and chitosan are common substrates of cellulase, hemicellulase, amylase, lipase, protease, and lysozyme enzymes, producing oligosaccharides as hydrolysis products under ambient conditions. These enzymes are available in bulk quantities at low cost [32]. The preferences of lysozyme, cellulase, biopectinase, pancreatin, bromelain, papain and lactase for chitin and chitosan as substrates were examined by in-gel activity assays. Lysozyme and bromelain preferred chitin as a substrate (Figure 2A), while cellulase possessed high hydrolytic activity for chitosan as a substrate (Figure 2B). Pancreatin was able to degrade both chitin and chitosan to an impressive degree, producing a clear zone not only around itself but a slightly clear region along its migration track from MW 94 to 67 during electrophoresis (Figure 2). This result reflected the fact that pancreatin is an SDSR enzyme and implied that the pancreatin is a potent exochitinase and its structure is rigid. Usually, enzymes with this type of more compact conformation are more stable and have longer life spans, and they are less affected by environmental factors [33,37]. And the products of exochitinase are uniform. Therefore, pancreatin is a good candidate for industrial-scale production of homogeneous chito-oligosaccharides.

### 2.3. Characteristics of Pancreatin

“Pancreatin” is a combination of digestive enzymes that are secreted by the pancreas. Pancreatin possesses amylase, trypsin, lipase, ribonuclease and protease activities, though only the amylase, protease and lipase activities are specified in the National Formulary and the U.S. Pharmacopeia. However, pancreatin has not previously been reported to act on chitin or chitosan. The molecular weights of the proteases, amylases and lipases in pancreatin are 23.8 kDa, 45 kDa and 38 kDa, respectively. The functions of most of the proteins in pancreatin are still unclear.

The substrate specificity of pancreatin was examined using *p*-nitrophenol (PNP)-modified GlcNAc derivatives at pH 5. Of the substrates tested, PNP-(GlcNAc)_2_ was the most sensitive to pancreatin, associated with a specific activity of 0.1949 U/mg (Table 1). This result indicated that pancreatin is a chitotriosidase and showed that pancreatin has no *N*-acetylglucosaminidase activity. 

**Table 1:** Substrate specificity of pancreatin.

Substrate	Specific activity (U/mg)
PNP-GlcNAc	0
PNP-(GlcNAc)_2_	0.1949 ± 0.0072
PNP-(GlcNAc)_3_	0.1534 ± 0.0099

**Figure 3:**
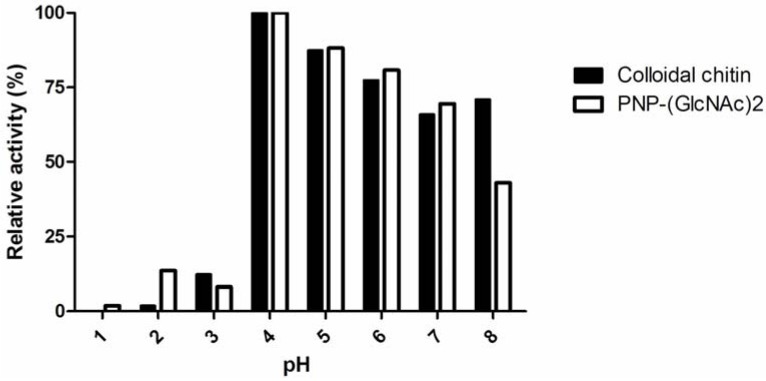
The effect of pH on the chitinase and chitotriosidase activities of pancreatin.

Two types of chitinase, acidic mammalian chitinase (AMCase) and chitotriosidase, have been identified in mammals [28]. AMCase is most active at pH 2, while chitotriosidase is most potent at pH 4–7. We identified the optimal pH for chitin derivative degradation by pancreatin using colloidal chitin and PNP-(GlcNAc)_2_ as substrates at different pH values. The activity of pancreatin peaked at pH 4 (Figure 3). This result confirmed that pancreatin possesses a chitinase activity, resembling chitotriosidase.

**Figure 4:**
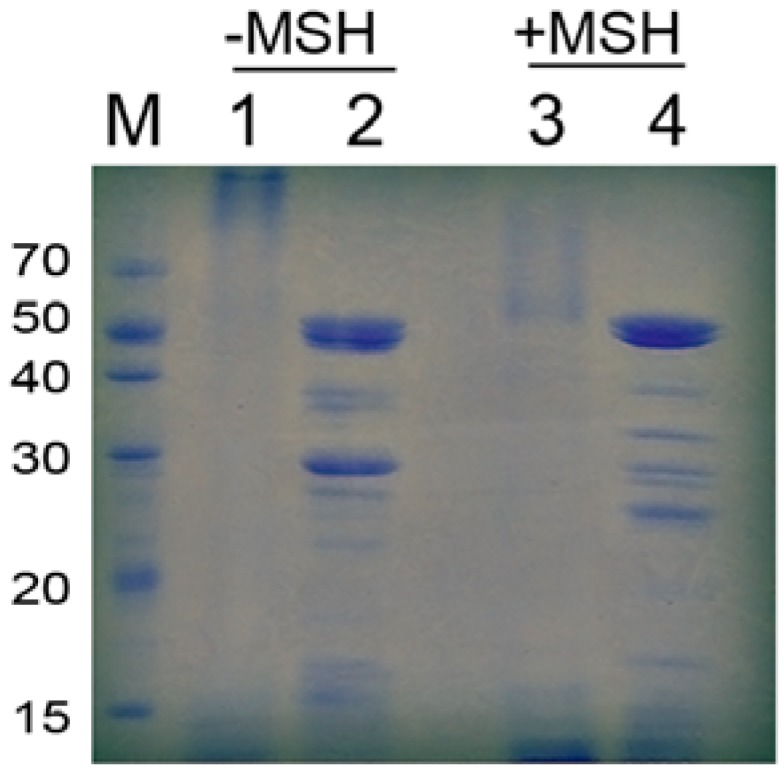
Pancreatin analyzed on an SDS-PAGE gel with or without MSH. Lanes 2 and 4 were heated before SDS-PAGE analysis.

As evaluated by SDS-PAGE, pancreatin exhibited a high molecular weight and multiple protein bands (Figure 4). The patterns observed for pancreatin treated with heat, 2-mercaptoethanol (MSH) or both were distinct, indicating that pancreatin is a complex composed of many proteins that are linked by both covalent and non-covalent bonds. The interaction forces identified were disulfide bonds, hydrophobic interactions, hydrogen bonds, electrostatic forces and van der Waals forces.

**Figure 5:**
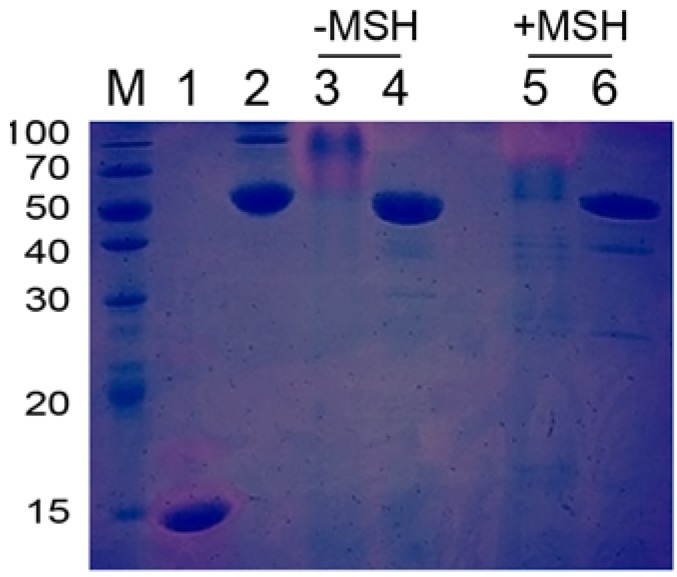
Pancreatin analyzed using the in-gel activity assay with a glycol chitin substrate. Lane 1, lysozyme, as positive control; Lane 2, bovine serum albumin, as negative control; Lane 3, pancreatin; Lane 4, heated pancreatin; Lane 5, pancreatin treated with MSH; Lane 6, heated pancreatin treated with MSH.

The differentially treated pancreatins described above behaved differently in the in-gel chitinase assay. Chitinase activity was only present in the MSH-treated pancreatin. However, the chitinase activity was lost upon heating (Figure 5). This result indicated that the chitinase in the pancreatin complex consists of non-covalently bonded subunits. In addition, the enzyme was thermal unstable.

Finally, two major forms of chitin, α-chitin and β-chitin, were used as substrates of pancreatin for a pilot trial of its industrial application. α-Chitin is the more abundant form. The structure of α-chitin is more compact, crystalline and rigid than that of β-chitin because it contains more hydrogen bonds. As with other chitinases, pancreatin hydrolyzed β-chitin more easily than α-chitin (Table 2).

**Table 2:** Specific activity of pancreatin for degrading different types of chitin.

Substrate	Specific activity (U/mg)
α-chitin	0.0154 ± 0.0019
β-chitin	0.0337 ± 0.0019

## 3. Experimental

### 3.1. Materials

Hog pancreatin was purchased from Biocon Ltd. (Nagoya, Japan), and was prepared as described previously [39]. The extracts were dialyzed using a 12K-14K molecular cut-off membrane in 20 mM Tris-HCl, pH 8. After centrifugation, the supernatant was used as pancreatin. Chemicals were obtained from Sigma-Aldrich (St. Louis, MO, USA).

### 3.2. Preparation of Glycol Chitin and Glycol Chitosan

Glycol chitin and glycol chitosan were prepared as previously reported [21]. Briefly, powdered chitin or chitosan (5 g) were mixed with 42% NaOH (100mL) for 4 h. A colloidal suspension was isolated by adding the mixture to ice (70 g), followed by filtration. Then, the suspension was mixed with 14% NaOH on ice (83 mL). Subsequently, ethylene chlorohydrin (50 mL) was added, and the mixture was stirred for 30 min. Glycol chitosan was obtained after overnight incubation at room temperature. Glycol chitin was produced by addition of acetic anhydride (10 mL) after overnight incubation. The products were further purified by dialysis and lyophilization.

### 3.3. In-gel Assays of Chitin or Chitosan

The concentrations of glycol chitin and glycol chitosan added to the SDS-PAGE gels were 0.01% and 0.005%, respectively. After electrophoresis, the polyacrylamide gel was soaked in 100 mM sodium acetate buffer, pH 5, with 1% Triton X-100 at 37 °C for 3 h. Subsequently, the gel was stained with CBB R-250.

### 3.4. Chitinase Activity Assay

The colloidal chitin substrate was prepared as previously reported [33,35,40]. The enzyme was incubated with 3 mg/mL substrate in 0.2 M potassium phosphate buffer at 37 °C for 30 min at the indicated pH. After the reaction, the absorbance with potassium ferricyanide, was measured at 420 nm. One enzyme unit was defined as the amount of enzyme that produced 1 μmol of reducing sugars in 1 min.

### 3.5. Substrate Specificity Assay

Substrates (0.18 mM) were mixed with enzyme at 30 °C for 30 min at the indicated pH. The reaction was terminated by the addition of 100 mM NaOH. The absorbance was measured at 405 nm [22]. One enzyme unit was defined as the amount of enzyme that produced 1 μmol PNP in 1 min.

## 4. Conclusions

A platform for screening chitinolytic and chitosanolytic enzymes was established. Using this platform, chitinase activity was identified in the high molecular weight subunits of the hog pancreatin complex for the first time. Pancreatin may be classified as a chitotriosidase based on its substrate specificity and optimal pH.
